# A novel upper-limb tracking system in a virtual environment for stroke rehabilitation

**DOI:** 10.1186/s12984-021-00957-6

**Published:** 2021-11-27

**Authors:** Kuan Cha, Jinying Wang, Yan Li, Longbin Shen, Zhuoming Chen, Jinyi Long

**Affiliations:** 1 College of Information Science and Technology and Guangdong Key Laboratory of Traditional Chinese Medicine Information Technology, Jinan University, Guangzhou, 510632 China; 2 Department of Rehabilitation Medicine, The First Affiliated Hospital of Jinan University, Guangzhou, China; 3 Pazhou Lab, Guangzhou, 510335 China

**Keywords:** Virtual reality, Avatar control, Rehabilitation, Upper limb, Motor function

## Abstract

**Background:**

The transfer of the behaviors of a human’s upper limbs to an avatar is widely used in the field of virtual reality rehabilitation. To perform the transfer, movement tracking technology is required. Traditionally, wearable tracking devices are used for tracking; however, these devices are expensive and cumbersome. Recently, non-wearable upper-limb tracking solutions have been proposed, which are less expensive and more comfortable. However, most products cannot track the upper limbs, including the arms and all the fingers at the same time, which limits the limb parts for tracking in a virtual environment and may lead to a limited rehabilitation effect.

**Methods:**

In this paper, a novel virtual reality rehabilitation system (VRRS) was developed for upper-limb rehabilitation. The VRRS could track the motion of both upper limbs, integrate fine finger motion and the range of motion of the entire arm and map the motion to an avatar. To test the performance of VRRS, two experiments were designed. In the first experiment, we investigated the effect of VRRS on virtual body ownership, agency and location of the body and usability in 8 healthy participants by comparing it with a partial upper-limb tracking method based on a Leap Motion controller (LP) in the same virtual environments. In the second experiment, we examined the feasibility of VRRS in upper-limb rehabilitation with 27 stroke patients.

**Results:**

VRRS improved the users’ senses of body ownership, agency, and location of the body. The users preferred using the VRRS to using the LP. In addition, we found that although the upper limb motor function of patients from all groups was improved, the difference between the FM scores tested on the first day and the last day of the experimental group was more significant than that of the control groups.

**Conclusions:**

A VRRS with motion tracking of the upper limbs and avatar control including the arms and all the fingers was developed. It resulted in an improved user experience of embodiment and effectively improved the effects of upper limb rehabilitation in stroke patients.

***Trial registration*:**

The study was registered at the First Affiliated Hospital of Jinan University Identifier: KY-2020–036; Date of registration: June 01, 2020.

**Supplementary Information:**

The online version contains supplementary material available at 10.1186/s12984-021-00957-6.

## Introduction

Stroke is a common health-care problem that results in obvious muscle weakness on one side of the body [1]. It is estimated that 50% to 75% of stroke patients have persistent impairment of the affected upper limb and must undergo repetitive physical training to recover their motor function [2]. To provide a more enjoyable and personalized motor rehabilitation experience, the use of virtual environments (VEs) as a tool is gradually becoming popular in the field because it offers richness of experience and is interesting to patients [3]. The virtual nature of the environment allows behaviors that are impossible or very expensive in reality to be implemented in a low-cost way. Bortone et al. [4–6] found that the use of a virtual environment and wearable devices offers a viable alternative to conventional therapy for improving upper extremity function in children with neuromotor impairments. Additionally, previous works indicate that the strength and ability of the affected side of the body can be effectively improved during rehabilitation that involves controlling an avatar’s upper limbs to interact with objects in VEs [7].

In many applications, avatars are used as the interface that allows people to interact with VEs [8]. Movement tracking is one of the key technologies for avatar control [9]. For partial upper-limb tracking, consumer devices, including hand-held controllers such as Oculus Touch or HTC VIVE controllers, and motion sensing devices, such as Kinect and Leap Motion controllers, are widely used [10]. Granqvist et al. [11] used HTC VIVE controllers with inverse kinematics for partial upper-limb tracking. However, the hand-held controller can only track the position of the hand and cannot provide information about the position of the fingers. Collingwoode-Williams et al. [12] built a system to study the effect of lip and arm synchronization on the feeling of body ownership. They used a Kinect device for body tracking and an Oculus Rift device for head rotation measurement. However, the Kinect device can only track the arms and a few key points on each hand (one key point on the fingertip, one key point on the thumb, and one key point on the whole hand). Complete upper-limb tracking enhances the realism of an avatar’s upper-limb behavior, which influences patients’ cognition and may be beneficial for rehabilitation [13]. Generally, for high-quality upper-limb tracking including the arms and all the fingers, it is necessary to use marker-based tracking systems that require the user to wear tracking suits, such as that employed by the OptiTrack system [13, 14]. These suits are expensive and cumbersome. The use of consumer devices is another less expensive solution. Lin et al. [15] used the Oculus Rift headset with a Kinect sensor, a Leap Motion controller and a dance pad to allow users navigate and manipulate objects inside synthetic scenes. With this method, only one Kinect was used, and it could not recognize which side of the body was facing the device. Wu et al. [16] introduced a setup that integrated multiple Kinects for robust and accurate full-body 3D skeleton tracking and a Leap Motion controller for tracking the hands. However, the tracking area of the Leap Motion controller was limited to a small range above the device.

In this paper, we propose a method that can control avatars’ upper limbs by tracking the movements of both arms and all fingers in a large tracking space. Using this method, we developed a virtual reality rehabilitation system (VRRS) that can map the movement of a user’s upper limbs to an avatar’s upper limbs to improve the user’s cognitive and affective experience during interaction in VEs. The difficulty of tasks can be conveniently modified to dynamically match the patient’s motor function, which is important for motor learning in general [17] and for rehabilitation in particular [18]. Hence, we hypothesized that VRRS can provide an effective rehabilitation training method to improve stroke patients’ recovery of their upper-limb motor function by enhancing their sense of body ownership, agency, and location of the body.

To test our hypothesis, we first performed an experiment with 8 healthy participants to compare VRRS with the partial tracking method based on Leap Motion [19]. In the experiment, we built VEs customized for upper-limb motion and assessed the senses of body ownership, agency, and location of the body and the usability of the systems. Then, to evaluate the feasibility of using the VRRS for rehabilitation training, another experiment was performed. For that experiment, 27 stroke patients were recruited, and the Fugl-Meyer (FM) scores for the patients’ upper limb motor functions were evaluated.

## Methods

### System architecture

**Fig. 1:**
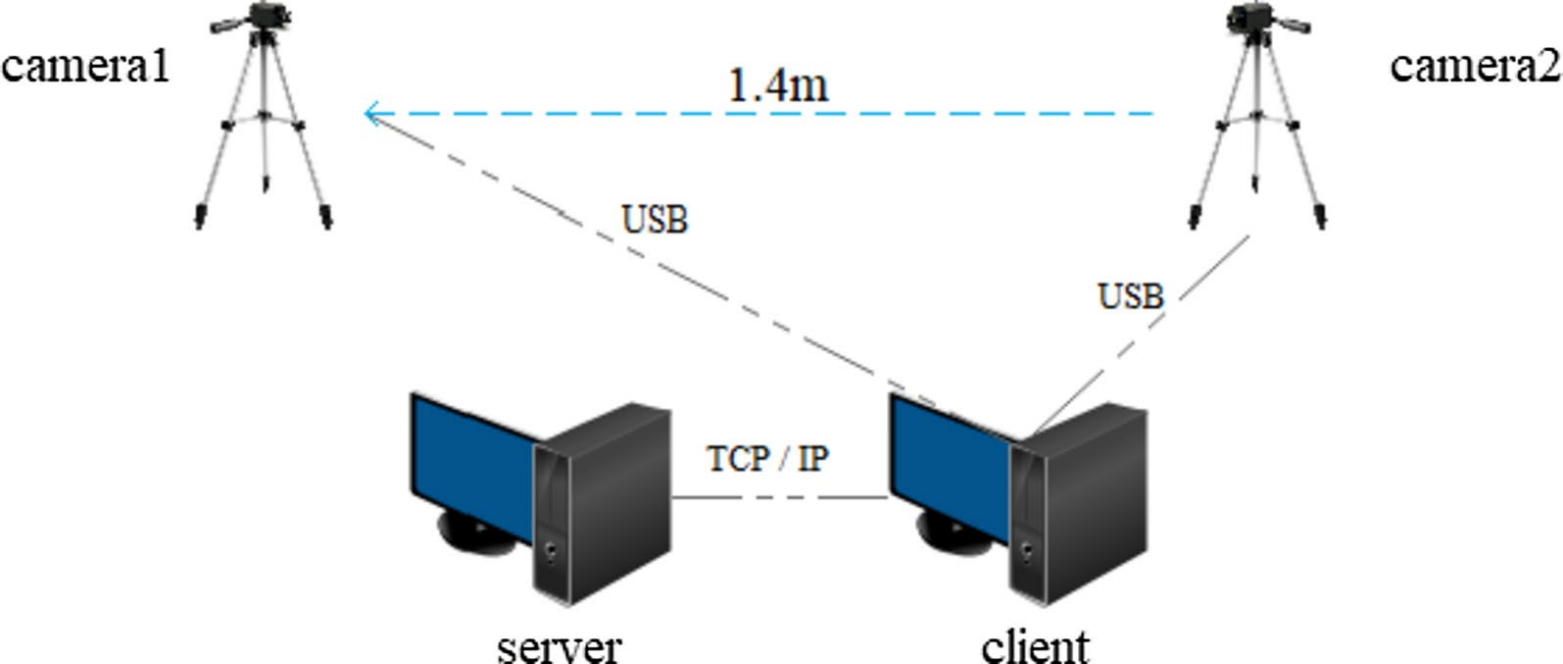
VRRS setup including two computers and two cameras

As shown in Fig. 1, the VRRS consists of two cameras (BFS-U3–13Y3C-C, FLIR Systems, Inc.) and two computers, including a client computer (Windows 8, Intel Core i7–6700 at 3.40 GHz, and 8 GB RAM) and a server computer (Linux, Intel Core i9–9900 k at 3.60 GHz and 32 GB RAM and RTX2080TI). The cameras are fixed on two tripods placed on a table, which positions the cameras approximately 2 m above the ground. The distance between the two cameras is approximately 1.4 m, and they are connected to the client computer through USB cables.

The video data acquired by the two cameras are transferred to the server in real time. Then, the data are input to the OpenPose platform configured on the server for processing to recognize the tracked person’s pose. The computer with Unity (2018.3.12f1) is the client which receive the data of the positions of 6 joints of the arms and 42 joints of the fingers from the server through TCP/IP network architecture customized in C#. Afterward, the position data for the joints are used to synchronize the action of the user's avatar.

At each time step, the sensor data transferred from the OpenPose platform are used to establish the avatar's pose. The OpenPose platform can be used to track the movement of people within a wider space than the Leap Motion controller and has great robustness for accurately estimating a person’s pose, even if part of his or her body is occluded. The sensor data received by the server computer are reorganized into a series of 3D coordinate data. Then, the 3D data are filtered and used to obtain joint orientation data. The orientation of the finger joints is used to set the avatar’s fingers with forward kinematics, which can simulate finger movements naturally. The position of the arm joints is applied to the avatar’s arms with inverse kinematics based on an analytic method. Using inverse kinematics for arms provided a better experience than using forward kinematics during the performance of the relatively simple motor tasks used in this study. As a result, the avatar's upper limbs, including all fingers and the entirety of both arms, can be controlled for interaction with the VEs.

### Study

**Fig. 2:**
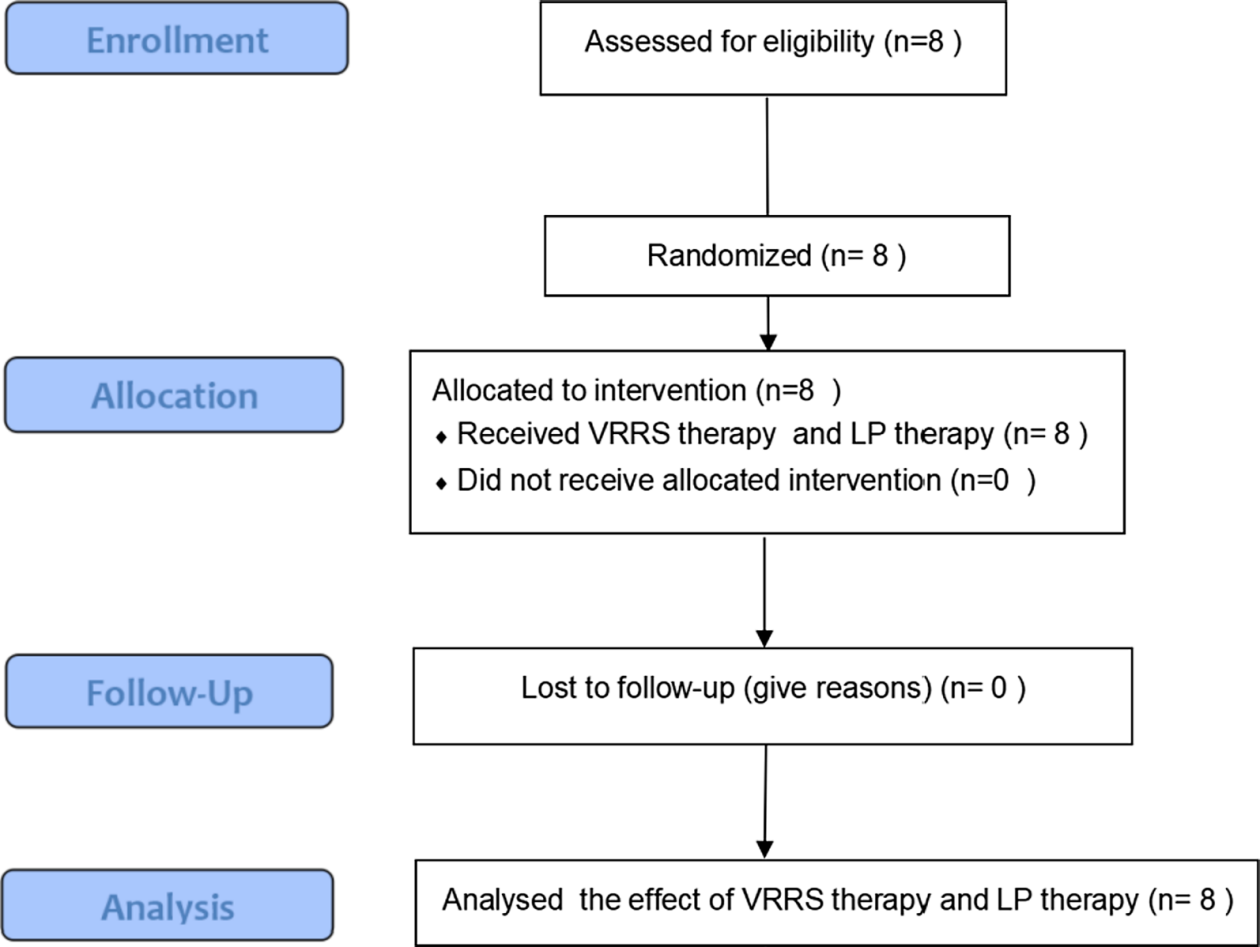
CONSORT flow diagram of the first experiment. CONSORT flow diagram illustrating participant flow during the different phases of the experiment

**Fig. 3:**
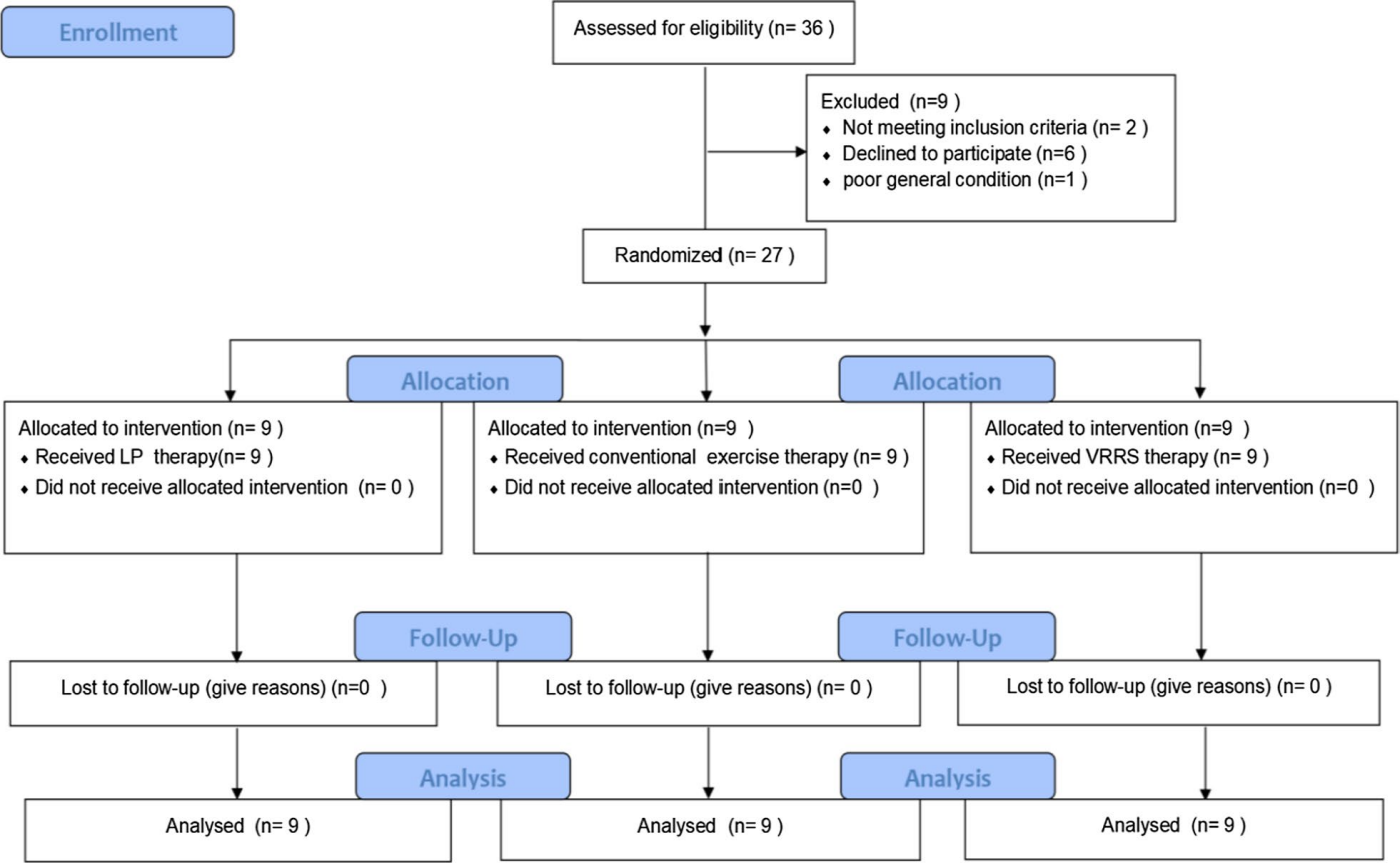
CONSORT flow diagram of the second experiment. CONSORT flow diagram illustrating participant flow during the different phases of the experiment

The main purpose of this study was to test the effect of the VRRS and evaluate its feasibility for rehabilitation training. In the study, two experiments were performed. Figures 2 and 3 provide overviews of the two experiments according to the CONSORT statement. In the first experiment, 8 participants were recruited to examine the effects of the VRRS and Leap Motion methods on virtual body ownership, agency, location of the body and usability. Twenty-seven stroke patients who had undergone occupational and physical therapy participated in the second experiment. Nine of these patients used the VRRS for rehabilitation training, 9 others underwent rehabilitation training with the LP system, and the remaining 9 patients received conventional therapy.

### Participants

A total of 35 volunteers participated in the study: 8 healthy subjects (4 male, 4 female) aged 21–26 years (M = 22.6, SD = 1.8) who were recruited from our university through advertisements posted on a social media platform, and 27 stroke patients (15 male, 12 female) with normal vision who were aged 10–90 years (M = 45.8, SD = 21.6). The 27 patients were randomly assigned to three groups. Nine patients without severe cognitive impairment formed the experimental group and performed the tasks in Experiment 2 using the VRRS. Nine other patients composed Control Group 1 and completed the tasks in Experiment 2 using conventional therapy methods. The remaining 9 patients completed the tasks in Experiment 2 using the LP and served as Control Group 2. These patients had different levels of disability, with Fugl-Meyer [20] scores ranging from 4 to 58 (M = 24.0, SD = 17.8). The patients gave their informed consent to participate in the experimental procedures, which were approved by the local ethics committee at Jinan University and were in accordance with the guidelines established in the Declaration of Helsinki. In our first experiment, each subject completed each of the two treatment-phases in a double-blind, randomized order. Each treatment-phase involved three kinds of tasks. The 8 healthy subjects were randomly allocated by using randomization codes.

## Experimental procedure

### Experiment 1

**Fig. 4:**
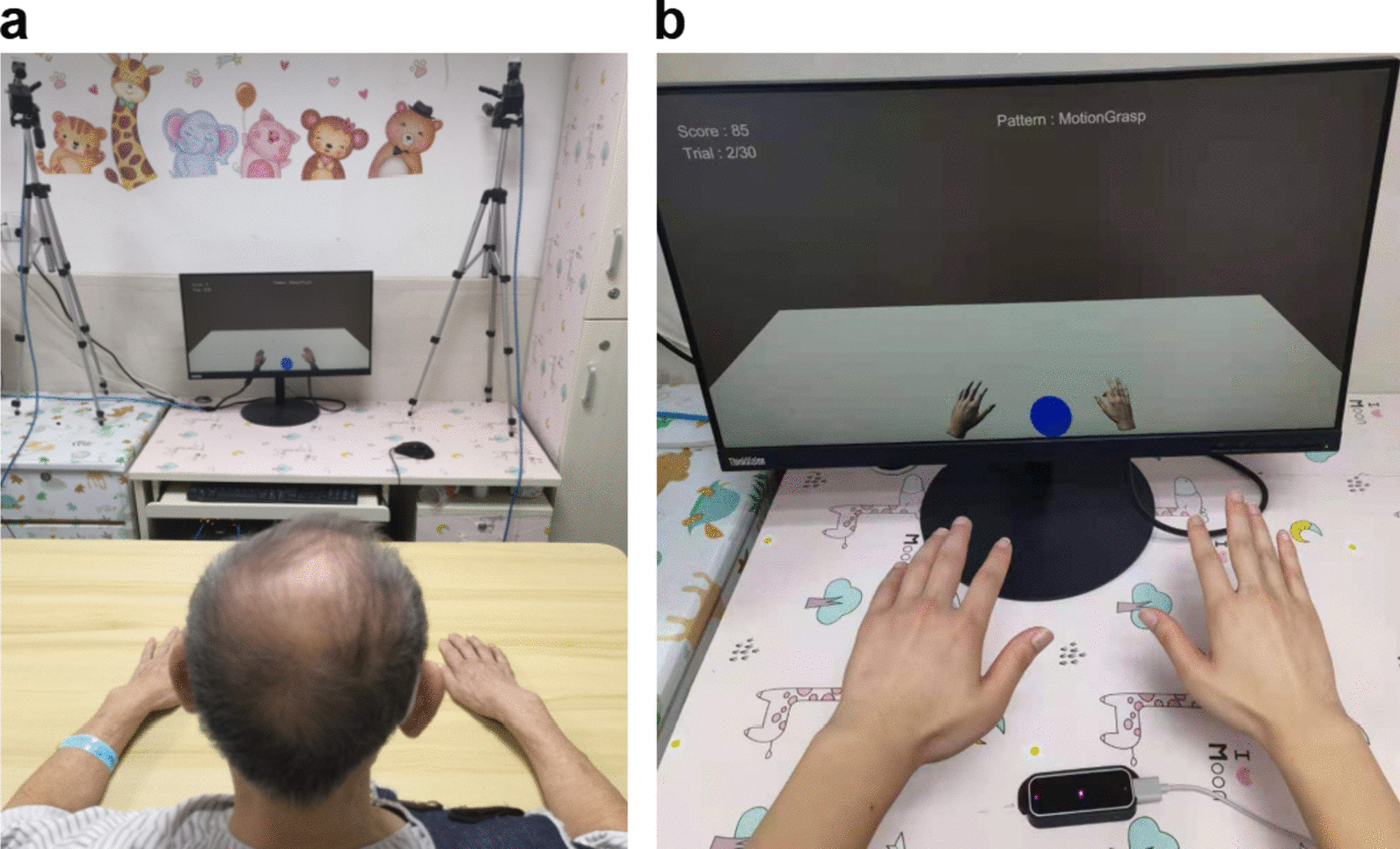
Participants performing experiments. **a** A patient performing experiment 2 with VRRS. **b** A healthy subject performing experiment 1 with LP

In the experiment, the subjects performed three kinds of tasks in VEs—normal motion on a plane, mirrored motion on a plane, and grasping motions—with the VRRS and LP systems; i.e., six tasks were performed (Fig. 4b). The normal motion task and the grasping motion task are conventional motor tasks for rehabilitation [21] and the mirrored motion task which is an emerging therapy can promote brain function remodeling and induce motor function recovery [22]. Before each task started, the subjects were positioned in a chair which was approximately 1.6 m away from cameras with an initial upper body posture that was the same as that of the avatar. Their hands were stretched naturally, with palms facing down. Each task consisted of 30 trials, and the six tasks took approximately 45 min. After all tasks were implemented, the subjects were asked to fill in a questionnaire (see Additional file 1) to grade both the VRRS and LP systems.

#### Task 1/Task 3: Normal motion on a plane based on the VRRS/LP

**Fig. 5:**
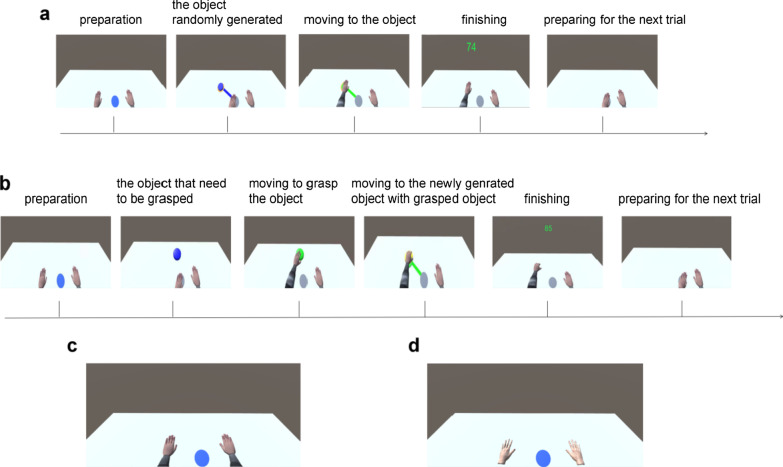
**a** One trial of left upper limb during task 1/3. **b** One trial of left upper limb during task 5/7. **c** An avatar with hands and arms as the representation of the user using VRRS. **d** An avatar only with hands as the representation of the user using LP

The paradigms of one trial were based on the experimental procedure presented in Fig. 5a. Initially, the subject controlled the avatar to move its left upper limb/virtual left hand to the starting area, which was originally displayed in blue, by moving his or her left upper limb. The subject held this position for 1.5 s, after the area turned green. After that, a blue object of a constant size was generated and located in a random location in the reaching area, which was calibrated for each subject before the experiment began. The color of the starting area turned gray, and a blue line appeared between the object and the starting area. The subject was required to move his or her left upper limb along the blue line to reach the object and hold it for 1.5 s after the object and the line turned green. If the object could not be reached within 4 s, both it and the line disappeared, and the trial was regarded as unfinished. At that point, the subject had to move his or her left upper limb as quickly as possible back to the starting area. After he or she had returned to the starting area and the color of the starting area returned to blue, the next trial began. In contrast, if the subject was able to reach the object, a score was displayed on the top-middle of the screen for 1 s to provide encouraging visual feedback. The total score was shown in the upper-left corner of the screen throughout the task, which also served as visual feedback. Then, the subject moved the left upper limb back to the starting area as soon as possible and remained there until the starting area turned blue again, which meant that the trial had been completed and the next trial was beginning. The first 15 trials used the left upper limb; the remaining trials used the opposite limb, with the subject controlling the movements of the avatar's right upper limb/virtual right hand in the same manner (Fig. 5).

#### Task 2/Task 4: Mirrored motion on a plane based on the VRRS/LP

In these two tasks, the motion of the subject’s left upper limb was mapped to the avatar's right upper limb/virtual right hand. The avatar's left upper limb/virtual left hand was controlled by the subject’s opposite limb. Other procedures were the same as those used in task 1 and task 3.

#### Task 5/Task 6: Grasping motion based on the VRRS/LP

First, after the subjects moved their left upper limbs/virtual left hands to the starting area, blue object 1 was randomly generated in the reaching space. The reaching space was determined by the subject’s reach area and constant height. Then, the subject was required to grasp the object and hold it for 1.5 s after the object turned green. If the object could not be grasped within 4 s, it disappeared, and the task was regarded as unfinished. If the object was grasped, it turned yellow, and a new blue object 2 was randomly generated in the reaching area after 1.5 s. At the same time, a blue line appeared that connected object 2 and a point on the same plane that was based on the generated position of object 1. The subjects were asked to move their left upper limbs to reach object 2 along the blue line (Fig. 5). Other procedures were the same as those used in task 1 and task 3.

### Experiment 2

**Fig. 6:**
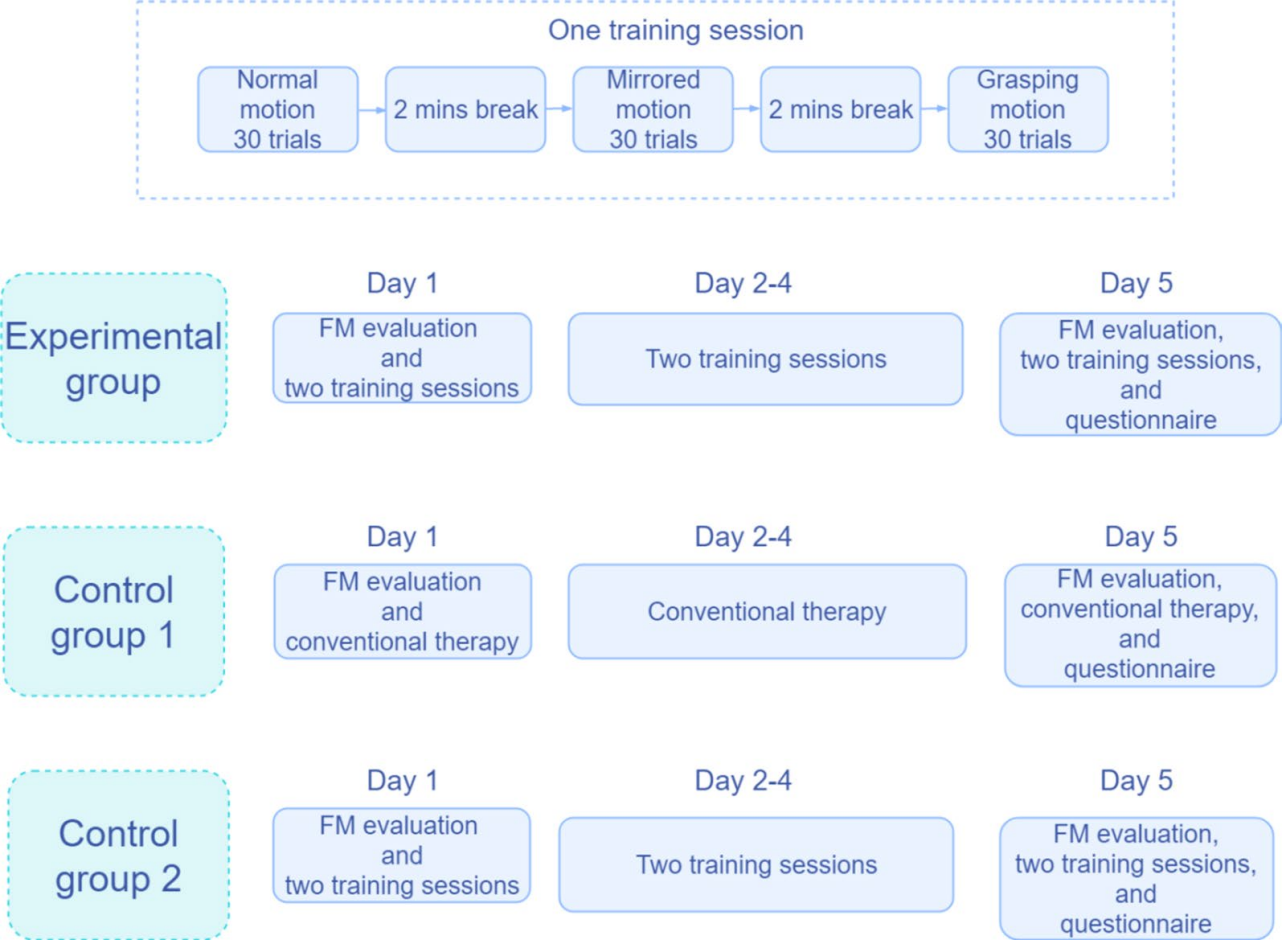
Design of the experiment 2

Experiment 2 was a 5-day experiment that was completed by the subjects in all three groups with the same training time (350 min, 70 min per day). All patients were evaluated with FM on the first day and evaluated again with FM after they had completed the 5-day experiment. The patients in the experimental group and Control Group 2 were also asked to fill out a questionnaire after completing the experiment. Every stroke patient in the experimental group participated in the experiment on the feasibility of the VRRS (Fig. 4a), which included ten training sessions. These patients were required to participate in sessions twice a day with an interval of 2 min between sessions. Before the first session, the motor function of the stroke patients was evaluated with FM. Each session consisted of three tasks: normal motion on a plane, mirrored motion on a plane, and grasping motion. Based on the duration and intensity that the patients were able to adapt to, the number of trials for each task was set to 30, and the patients took 2-min breaks between the three tasks. In Control Group 1, the patients received conventional therapy for shoulder, elbow, wrist, and finger mobilization. In Control Group 2, the patients performed the same training procedure as the experimental group, but with LP (Fig. 6).

### Outcome measures

In Experiment 1, the variables (unfinished rate, game performance, and velocity peaks of the wrists) were measured by Unity for each task. The unfinished rate was obtained by dividing the quantity of unfinished trials by the total number of trials in each task. The velocity peaks were measured as the subjects moved along the line in the VEs. Game performance was determined by the total score, which was the sum of the scores for 30 trials. The score for each trial was determined by the average distance between the line and the location based on the position of the center of the hand. The location is obtained by mapping the position of the hand to a plane at the same height as the line. The score for each trial was calculated as follows:

$${\text{Score}} = \left( {0.1 - {\text{average}}\,{\text{distance}}} \right)*1000,$$

where the maximum score is 100, and the average distance denotes the mean value of the sum of the distance between the location based on the hand position and the line in each frame during movement along the line. After the subjects completed all tasks, they were asked to complete the questionnaire, which integrated avatar embodiment [23] and a System Usability Scale [24]. The questionnaire was used to assess the sense of body ownership, agency, and location of the body and the usability of the VRRS and LP. In Experiment 2, the 24 patients underwent two FM evaluations. The patients in the experimental group and Control Group 2 were also asked to fill in the same questionnaire that they had completed in Experiment 1 after the second evaluation.

### Statistical analysis

Analysis was performed using the SPSS statistical software system (version 25.0) and the Origin function draw tool (version 2018). First, we used a homogeneity of variance test to determine whether the overall variance was unequal. Only the unfinished rate for the normal motion and grasping motion tasks were not consistent with homogeneity of variance. Then, in Experiment 1, we used the analysis of variance of two-factor fixed design to compare the VRRS and LP in terms of game performance, velocity peaks, unfinished rate, and the participant responses to the questionnaire. In Experiment 2, the paired t-test was applied to compare the FM scores at the two evaluations for each group. For the participants’ responses to the questionnaire, we used an unpaired t-test to examine differences. We also used one-way repeated measure ANOVA to separately compare the FM scores at the first evaluation, those at the second evaluation, and the D-value of the FM scores at the two evaluations among the three groups. The significant values were considered as p < 0.05.

## Results

### Experiment 1

The results of the survey performed at the end of the first experiment are shown in Table 1. For location of the body, agency, body ownership, and usability, there were significant differences between the VRRS and LP. In addition, the mean values of these variables were higher when the VRRS was used than when LP was used. The participants’ responses to the VRRS were generally quite positive. Five of eight subjects agreed that the upper-limb behaviors of the avatar were authentic, as if the virtual upper limbs were parts of their bodies. In addition, 5 of 8 subjects indicated that they would like to continue to use the VRRS if the equipment were available.

**Table 1:** Results of the survey performed at the end of experiment 1

	VRRS	LP	p
Body ownership	2.2 (1.8)	0.4 (1.8)	0.049*
Agency	7.6 (2.2)	4.4 (4.1)	0.025*
Location of the body	3.8 (1.3)	2.5 (1.6)	0.01*
Usability	78.8 (8.2)	66.9 (14.1)	0.035*

Likert-scale from − 6 to + 6 employed for Location of The Body; Likert-scale from − 12 to + 12 employed for Agency; Likert-scale from − 6 to + 6 employed for Body Ownership; Likert-scale from 0 to 100 employed for Usability. Higher numbers denote more positive responses. Mean (SD), p < 0.05* using the analysis of variance of two-factor fixed design

The symbol [bold] means significant difference

The game performance, peak velocity, and unfinished rate data are presented in Table 2. Few statistically significant differences between the VRRS and LP were apparent for these three indices. However, the mean values of these three indices indicated better performance with the VRRS than with LP.

**Table 2:** Velocity peak, game performance, and unfinished rate under three conditions in experiment 1

	Grasping motion			Mirrored motion			Normal motion		
	VRRS	LP	p	VRRS	LP	p	VRRS	LP	p
Game performance	2223.1 (250.3)	2079.5 (277.3)	0.124	2139.9 (264.5)	2140.3 (226.5)	0.997	2366.8 (101.1)	2225.5 (271.0)	0.241
Peak velocity	0.99 (0.14)	0.95 (0.12)	0.513	1.08 (0.15)	1.01 (0.13)	0.484	1.19 (0.13)	1.04 (0.12)	0.046*
Unfinished rate	0.05 (0.05)	0.09 (0.09)	0.196	0.05 (0.05)	0.06 (0.06)	0.675	0.01 (0.02)	0.08 (0.10)	0.139

The maximum of Game Performance is 3000. Mean (SD), p < 0.05* using the analysis of variance of two-factor fixed design for game performance, velocity peaks, unfinished rate

The symbol [bold] means significant difference

#### Location of the body, agency, and body ownership

**Fig. 7:**
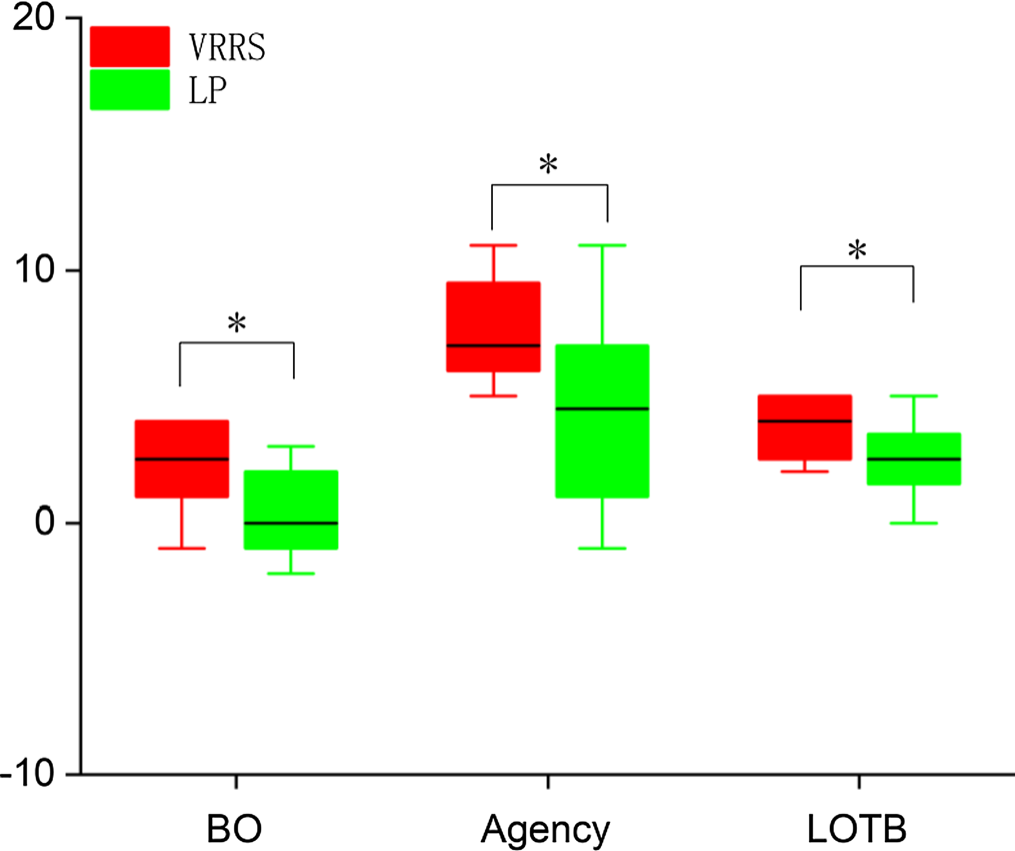
The results of responses for Location of The Body (LOTB), Agency, and Body Ownership (BO) in experiment 1. p < 0.05* using the analysis of variance of two-factor fixed design

Figure 7 shows the results from the body ownership (BO), agency, and location of the body (LOTB) sections of the questionnaire. The senses of body ownership, agency, and location of the body were measured with questions from an avatar embodiment questionnaire [23]. Note that the senses of location of the body, agency, and body ownership were enhanced with the VRRS compared with LP. There were significant differences (p < 0.05) between the VRRS and LP in terms of location of the body (p = 0.01), agency (p = 0.025), and body ownership (p = 0.049).

#### System usability

**Fig. 8:**
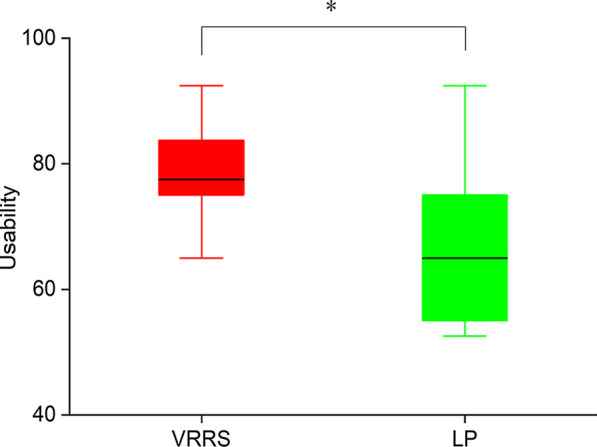
The results of participants responses for Usability in experiment 1. p < 0.05* using the analysis of variance of two-factor fixed design

In the experiment, system usability was measured with the System Usability Scale [24]. The figure shows that there was a significant improvement effect in terms of usability (p = 0.035) with the VRRS compared with LP (Fig. 8).

#### Velocity peak, game performance, and unfinished rate

**Fig. 9:**
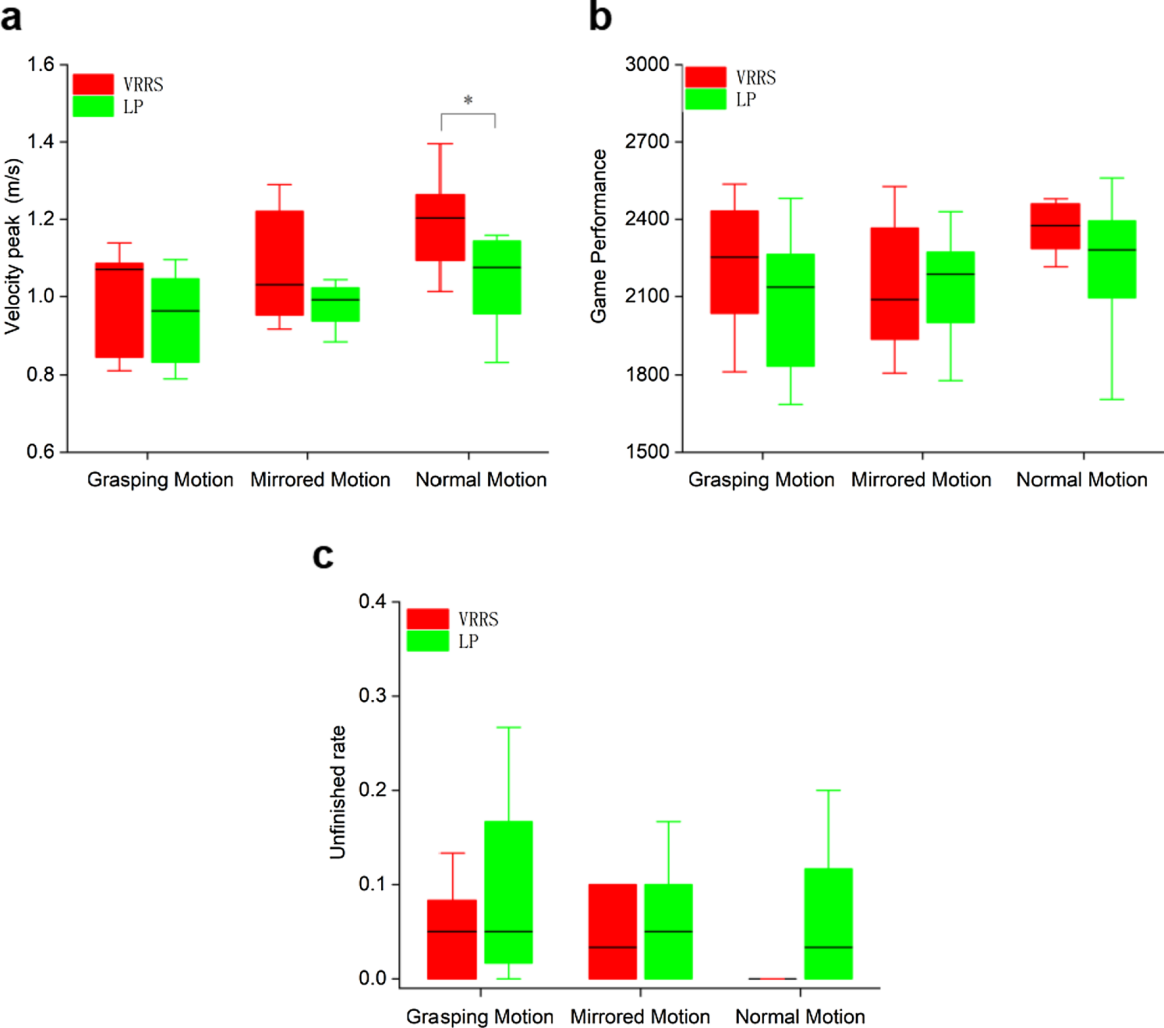
The results of Velocity peak, Game Performance, and Unfinished rate measured in tasks in experiment 1. **a** Velocity peak. **b** Game performance. **c** Unfinished rate. p < 0.05* using the analysis of variance of two-factor fixed design

The analysis of variance of two-factor fixed design was applied to measure the velocity peaks, game performance, and unfinished rate in various tasks. We found a significant difference in the velocity peak (p = 0.046) under normal motion conditions between the VRRS and LP. There were no significant differences between the VRRS and LP under the other conditions (Fig. 9).

### Experiment 2

In Experiment 2, we found significant differences between the first evaluation and the second evaluation in the experimental group (p = 0.000), Control Group 1 (p = 0.000), and Control Group 2 (p = 0.001). In addition, the mean D-value between the two evaluations was the highest for the experimental group (Table 3). The mean D-value of Control Group 2 was higher than that of Control Group 1.

**Table 3:** Results of FM for three groups in experiment 2

	Pre	Post	D-value	Pre vs post p-value
Control group 1	23.44 (18.94)	28.11 (19.48)	4.67 (2.24)	0.000*
Control group 2	27.67 (21.06)	33.56 (20.53)	5.89 (3.59)	0.001*
Experimental group	20.78 (13.97)	31.22 (15.55)	10.44 (5.32)	0.000*

Mean (SD), p < 0.05* using the paired t-test

The symbol [bold] means significant difference

Table 4 shows the results of the comparison of the first evaluation, the second evaluation, and the D-value between the three groups. There was a significant difference (F = 5.426, p = 0.005) with a large effect size ($\eta_{{\text{p}}}^{2}$ = 0.361) in the D-value between the experimental group and Control Group 1. In addition, there was also a significant difference (F = 5.426, p = 0.021) with a large effect size ($\eta_{{\text{p}}}^{2}$ = 0.221) in the D-value between the experimental group and Control Group 2.

**Table 4:** Comparison of FM scores between three groups in experiment 2

	Control group 1 vs control group 2 p-value	Control group 1 vs experimental group p-value	Control group 2 vs experimental group p-value	F-value
Pre	0.628	0.759	0.431	0.327
Post	0.541	0.726	0.793	0.193
D-value	0.515	0.005*	0.021*	5.426

p < 0.05* using one-way repeated measure ANOVA

The symbol [bold] means significant difference

The participants’ responses to the questionnaire were relatively positive. The participants indicated that they would like to continue to undergo rehabilitation training in VEs. The significant differences were found between the VRRS and LP in terms of usability (p = 0.038), agency (p = 0.049), and body ownership (p = 0.044). The mean values for location of the body, agency, body ownership, and usability were higher for the VRRS than for LP (Table 5).

**Table 5:** Results of the survey performed at the end of experiment 2

	VRRS	LP	p
Body ownership	3.2 (1.3)	1.3 (2.2)	0.044*
Agency	6.8 (2.0)	4.2 (2.9)	0.049*
Location of the body	4.2 (1.4)	3.6 (1.7)	0.371
Usability	69.7 (7.1)	55.8 (17.0)	0.038*

Likert-scale from − 6 to + 6 employed for Location of The Body; Likert-scale from − 12 to + 12 employed for Agency; Likert-scale from − 6 to + 6 employed for Body Ownership; Likert-scale from 0 to 100 employed for Usability. Higher numbers denote more positive responses. Mean (SD), p < 0.05* using the unpaired t-test

The symbol [bold] means significant difference

## Discussion

The findings of our first experiment indicated the impact of the VRRS on the interaction between the user and the virtual environment. The use of a depth sensor-based avatar control system has been shown to result in a higher sense of body ownership and agency than the use of controller-based avatar control system [25]. We showed that the VRRS could elicit higher feelings of body ownership, agency, location of the body and system usability, which supports the above hypothesis. According to the participants’ feedback, there were two reasons why the VRRS performed better than LP. The first reason was the VRRS’s full representation of users’ upper limbs in the VEs. The second reason was that the VRRS allows a wider range in which users can interact with VEs, which makes them feel more comfortable. We speculated that visual feedback that provides more complete and real presentation of behaviors in VEs can lead to a better sense of body ownership, agency, and location of the body.

This is in agreement with previous results that suggest that movement tracking and the representation of users in a virtual environment can affect the user’s sense of embodiment and interaction and their experience of space [26, 27]. Additionally, we found that only the velocity peaks under normal motion conditions were significantly different between the VRRS and LP. This might be because the difficulty and challenge of three different types of tasks affected the participants’ cognition and motion.

In our second experiment, we evaluated the feasibility of the VRRS for rehabilitation. The results showed that the VRRS promotes the effect of rehabilitation. In the comparison analysis, the FM scores of the experimental group, Control Group 1, and Control Group 2 were all significantly improved at the second evaluation and the mean D-values between the two evaluations for the experimental group were significantly higher than the other two groups. This might be because virtual reality programs are more interesting and enjoyable than traditional therapy [28], which improves patient engagement, and because the improved visual feedback provided by the more complete presentation of upper limbs behaviors in VEs can affect the patient's cognition and rehabilitation [13]. The effect of VEs on rehabilitation was also noted in previous studies. Saposnik et al. [29] found that VEs and video game applications are novel and potentially useful technologies for upper arm improvement after stroke. In addition, there is much evidence illustrating how VEs can be used as a therapeutic training tool that provides a learning experience tailored to individual clients [30]. We also found similar works indicating that tracking human movements in VEs is beneficial and necessary in rehabilitation schemes. For example, Lupu et al. [31] proposed an affordable motion tracking system for stroke recovery that involved placing markers on the patient’s limbs. Tsekleves et al. [32] developed and assessed an interactive game-based rehabilitation tool using a Microsoft Kinect sensor for balance training in adults with neurological injury. The results of these two experiments and our findings for the effect of a novel upper-limb tracking system in VEs and on rehabilitation may be the most important outcome of our study and may contribute significantly to rehabilitation research.

The study presents several limitations. First, VRRS provides only visual feedback without tactile feedback. This leads to a lack of touch, which may limit further improvement of the senses. Second, we only explore the effect of the whole VRRS including movement acquisition and visualization on the rehabilitation performance. It is interesting to explore which element of our VRRS system makes the improvement in the future. In addition, the motion patterns used in the VEs in the VRRS are simple and do not include more complex motor tasks, and motion tracking technology in our VRRS may need to be improved before more complex motion patterns added. It also needs to expand the sample size in the future work. Another aspect of the VRRS that could be improved is its assessment method. In VRRS, the patients’ upper limb motor function was evaluated solely through doctors’ professional judgment which may have a subjective impact on the evaluation results. Liao et al. [33] noted that patient contact with a clinician at every single rehabilitation session is economically unjustifiable, and they proposed a deep learning-based framework for the automated assessment of the quality of physical rehabilitation exercises. This intelligent assessment technology may be required in the further research.

## Conclusion

In this paper, we introduced a virtual reality rehabilitation system that could track the motion of the entirety of upper limbs and map that motion onto an avatar’s upper limbs. We also investigated the influence of the VRRS on body ownership, agency, location of the body and usability by comparing it with the LP system. Furthermore, we conducted an experiment with 27 stroke patients to evaluate the feasibility of using the VRRS in the rehabilitation of upper extremities. The results showed that the VRRS improves the user’s experience of body ownership, agency, and location of the body and can be employed to further optimize treatment.

In future work, we plan to explore the effect of the VRRS on more complex motor tasks and further optimize motion tracking technology to realize full upper-limb tracking more precise and improve engagement and transfer more real and natural behavior to the avatar during complex motor tasks. In addition, we will explore the respective contributions of motion acquisition and visualization to the improvement. Additionally, we are considering introducing tactile feedback into the VRRS and exploring the effect of multimodal feedback on interaction and rehabilitation. The intelligent assessment technology mentioned above also deserves research and we will expand the sample size in further study.

## Supplementary Information

**Additional file 1: Questionnaire.** Questionnaire administered following the VRRS system specific details.

## Data Availability

The data used in the current study are available from the corresponding author on reasonable request.

## Abbreviations

VRRS: Virtual Reality Rehabilitation System
LP: Leap Motion controller
VERGE: Virtual Environment for Rehabilitative Gaming Exercises system
FM: Fugl-Meyer;
VEs: Virtual environments
LOTB: Location of the body
BO: Body ownership
TCP: Transmission Control Protocol
