# Supplementary material for: A novel upper-limb tracking system in a virtual environment for stroke rehabilitation
Source: J Neuroeng Rehabil. 2021 Nov 27;18:166. doi: 10.1186/s12984-021-00957-6 (PMC8627064; doi:10.1186/s12984-021-00957-6)
Supplement: Supplementary file 1 — Additional file 1: Questionnaire. Questionnaire administered following the VRRS system specific details. [file 12984_2021_957_MOESM1_ESM.pdf]

**Body ownership(-3 - +3)**
**1.** I felt as if the virtual limbs/hands were my limbs/hands
**2.** It seemed as if I might have more than two limbs/hands
**Agency(-3 - +3)**
1. It felt like I could control the virtual limbs/hands as if they were my own limbs/hands
2. The movements of the virtual limbs/hands were caused by my movements
3. I felt as if the movements of the virtual limbs/hands were influencing my own movements
4. I felt as if the virtual limbs/hands were moving by themselves
**Location of the body(-3 - +3)**
1. I felt as if my limbs/hands were located where I saw the virtual limbs/hands
2. I felt as if my (real) limbs/hands were drifting toward the virtual limbs/hands or as if the virtual limbs/hands were drifting toward my (real) limbs/hands
Usability(1 - 5)
1. I think that I would like to use this system frequently
2. I found the system unnecessarily complex
3. I thought the system was easy to use

4. I think that I would need the support of a technical person to be able to use this system
5. I found the various functions in this system were well integrated
6. I thought there was too much inconsistency in this system
7. I would imagine that most people would learn to use this system very quickly
8. I found the system very cumbersome to use
9. I felt very confident using the system
10. I needed to learn a lot of things before I could get going with this system
